# Anterolateral congenital tibial bowing: case report

**DOI:** 10.3389/fped.2023.966358

**Published:** 2023-04-25

**Authors:** Giuseppe Mastantuoni, Angelo Gabriele Aulisa, Marco Giordano, Pietro Savignoni, Renato Maria Toniolo, Francesco Falciglia

**Affiliations:** ^1^ UOC of Traumatology, Bambino Gesù Children’s Hospital (IRCCS), Rome, Italy; ^2^ Department of Human Sciences, Society and Health, University of Cassino and Southern Lazio, Via S. Angelo in Theodice, Localita’ Folcara, Cassino, Italy

**Keywords:** congenital tibial bowing, congenital tibial pseudarthrosis, lower limb defects in children, congenital long bone curvatures, intramedullary tibial nail in children

## Abstract

**Background:**

The treatment of congenital curvatures (bowing) of the tibia still represents a challenging problem for all pediatric orthopedic surgeons because of its unpredictable course, especially if pseudoarthrosis occurs after a pathologic fracture of the tibia.

**Case presentation:**

We describe the case of a child affected by an isolated curvature of his left leg. The congenital malformation was discovered at birth and no other pathological clinical finding was present. The first x-ray showed the presence of a congenital curvature of the tibia of the antero-lateral type. He was born in another country (Romania) and when he first came to our clinical observation at the Orthopedic and Traumatology Department, Pediatric Hospital “Bambino Gesu’”, Rome, the child was 14 months of age and had already started walking. Only a leg discrepancy of about 2 cm was present with consequent pelvis obliquity. At the beginning, we prescribed external lower limb orthoses and a simple shoe rise to prevent a tibial pathologic fracture and reduce pelvic obliquity. At periodical clinical follow-up visits and despite the external lower limb orthoses prescribed, a progressive worsening of the severe congenital tibial curvature was observed together with signs and symptoms, such as pain and limping, that suggested an objective “pre-fracture stage” of the tibial curvature; we decided to perform surgery. At the time of surgery, the child was three and a half years old. Surgery consisted of a double osteotomy, both of the fibula and of the tibia. Subtraction of the distal meta-diaphyseal portion of the fibula and tibial osteotomy in **Correspondence:** of the major anterolateral curvature. The tibial osteotomy was then stabilized by an internal Rush rod inserted proximally to the tibia under the cartilage growth plate and made it end inside the distal tibial epiphysis, crossing the distal tibial cartilage growth plate, preserving the ankle joint.

**Results:**

The patient had an immediately excellent outcome. The tibial osteotomy site healed perfectly. At periodical orthopedic follow-up visits, the child was found to be always better. No clinical significative evidence of growth disturbances, due to the Rush rod that crossed the distal tibial cartilage growth plate, were noted. X-rays showed that the Rush rod progressively migrated with tibial growth together with the tibial bone growth, always getting further away from the distal tibial cartilage growth plate. Moreover, even the leg-length discrepancy and the pelvic obliquity improved. After an eight-year follow up, the patient, now a young boy of 11 and a half years, has an excellent outcome.

**Conclusions:**

Our case report undoubtedly provides further important information for the treatment of these rare congenital disorders. In particular, it highlights the management of the “pre-fracture stage” in a severe congenital tibial antero-lateral curvature in a very young child and describes the surgical technique performed.

## Introduction

Congenital bowing of the tibia is a rare condition, affecting one child every 140.000/190.000 births. It is usually noted at birth or shortly after and is considered the precursor of congenital pseudarthrosis of the tibia (CPT) (1, 2).

It is usually associated with genetic disorders such as neurofibromatosis type 1 (NF1), fibrous dysplasia, amniotic band syndrome, osteogenesis imperfecta, and bone fibromas (1).

Congenital bowing of the long bones can be more or less generalized or can regard a single bone segment, essentially the tibia point and in such last cases the condition is described as isolated curvature or “bowing” of the tibia. Several classification systems have been proposed to cover the spectrum of this condition; the most used is the one that describes three types of congenital curvatures of the tibia, referring to the convexity of the curvature always present at the distal third portion of the tibia: (1) antero-lateral, (2) posterior or posteromedial, and (3) anterior or anteromedial. The prognosis of these congenital disorders is very different (3).

The anterolateral bowing type is the one that often evolves into “secondary pseudarthrosis” of the tibia, although a benign form has been described (2–5). The prognosis of such congenital curvature must always be reserved since the surgical treatments are often very uncertain (4).

The posterior or posteromedial tibial curvature type is usually benign and presents a good prognosis. It is spontaneously regressive and surgical treatment can be considered without hesitation (4).

The anterior or anteromedial bowing type is often associated with congenital fibula defects, like aplasia or hypoplasia (6). The prognosis of this last type is also favorable.

The treatment for congenital curvatures of the tibia still represents a challenging problem for all pediatric orthopedic surgeons because of its unpredictable course if pseudarthrosis occurs after a pathologic fracture of the tibia.

Except for the resolving form, the natural history of anterolateral bowing, Crawford's type 2, is unfavorable if a fracture occurs and there is little tendency for the lesion to heal spontaneously despite the several surgical treatment options available, leading to amputation (5–7).

Because in the majority of cases, CPT is not present at birth, the term “congenital pseudarthrosis of the tibia” is somewhat inaccurate because only the underlying disease process and deformation of the tibia are usually present at birth, and is often just a question of time before a first fracture occurs (6).

Many treatment options are available for this disease, including both operative and non-operative options based on the severity of the condition (8–13).

Among the existing classification schemes that provide management guidance for these rare congenital disorders, Paley (2019) classified this condition based on severity, treatment, and prognosis (9).

In CPT, periosteal anomalies seem responsible for the curved dysplastic tibial bone that does not heal after a fracture. Thus, periosteal replacement addresses the pathogenesis of this disorder. As early as 1906, Codivilla proposed the concept of periosteal substitution (14, 15).

It is incredible that such a rare disorder still receives so much attention in the orthopedic literature. Pseudarthrosis of the tibia poses one of the most challenging treatment problems for all orthopedic surgeons because of the compounded difficulty of achieving and then maintaining union and simultaneously providing a functional extremity (6, 9).

There is a general pessimism as to the quality and longevity of any union that may be obtained, and the ultimate future function of the leg is uncertain. Although many treatment options exist, the fact that no one option has ever achieved long-lasting success with great frequency indicates that there is no single treatment for pseudarthrosis of the tibia (CPT) that will produce acceptable results in any predictable fashion (4, 9). The first step is to prevent fractures and give a normal alignment to the leg with or without fixation. There is no consensus on the appropriate age for surgery (16).

Initial treatment of tibial bowing deformity includes stretching, serial casting, or splinting. In many cases, a 50% correction of the deformity is usually observed by the age of two years, though a mild deformity often persists. Only significant deformity that interferes with growth may be an indication for tibial osteotomy, especially if little or no correction is seen by the age of two years or there is symptomatic and persistent deformity (17).

Many methods of treatment have been described for CPT, including mechanical (e.g., external fixators, nails), biological (e.g., free vascularized fibular graft, non-vascularized periosteum), and pharmacological (e.g., BMP, bisphosphonates) approaches as well as their combined use (18, 19). Together, these approaches have resulted in an amputation rate of 50% due to failure to achieve union in 20%–50% of cases and the occurrence of re-fractures in 30% of patients (18). The reported rate of bone union using several surgical techniques was 20%–50%, while bone union has been achieved in 100% of the cases treated with contralateral vascularized periosteal tibial graft transplantation (15). Longer follow-up for a refracture-free rate is needed to consider it safe enough to recommend it as a standard approach.

Another encouraging recently reported technique to treat CPT is the cross-union technique which also yielded excellent results with a 100% union rate with a seven-year mean follow-up (8, 9). Short-term treatment for CPT is a serious weakness for any study regarding this topic. Furthermore, in another recent paper, contrary to much of the established practice, osteotomies may be safely performed in CPT (19).

All these techniques represent new tools for the surgical treatment of these congenital disorders and these recent papers are showing excellent outcomes in obtaining bone union and reducing amputation rates for these conditions.

All together, furthermore, provide more information to all pediatric orthopedic surgeons who deal with such biologically complex situations and can finally start choosing the best reconstructive strategy for any of these singular congenital rare cases.

Current treatment protocols focus primarily on combining intramedullary fixation with external or internal fixation to achieve union rates between 74% and 100% (20). Intramedullary devices should be retained as long as possible to prevent refracture. Cross-union techniques, though technically difficult, have a reported union rate of 100% with no refracture at mid-long-term follow-up. Vascularized fibular grafting and induced membrane techniques can be successful but at the cost of numerous surgical procedures. Growth modulation is a promising new approach to preventing fractures altogether, though further study with larger patient series is necessary (15–22).

In this paper, we will limit the description and considerations on CPT and focus on our case report that contributes further significative information regarding the treatment and management of these rare congenital disorders.

## Case report

**Figure 1:**
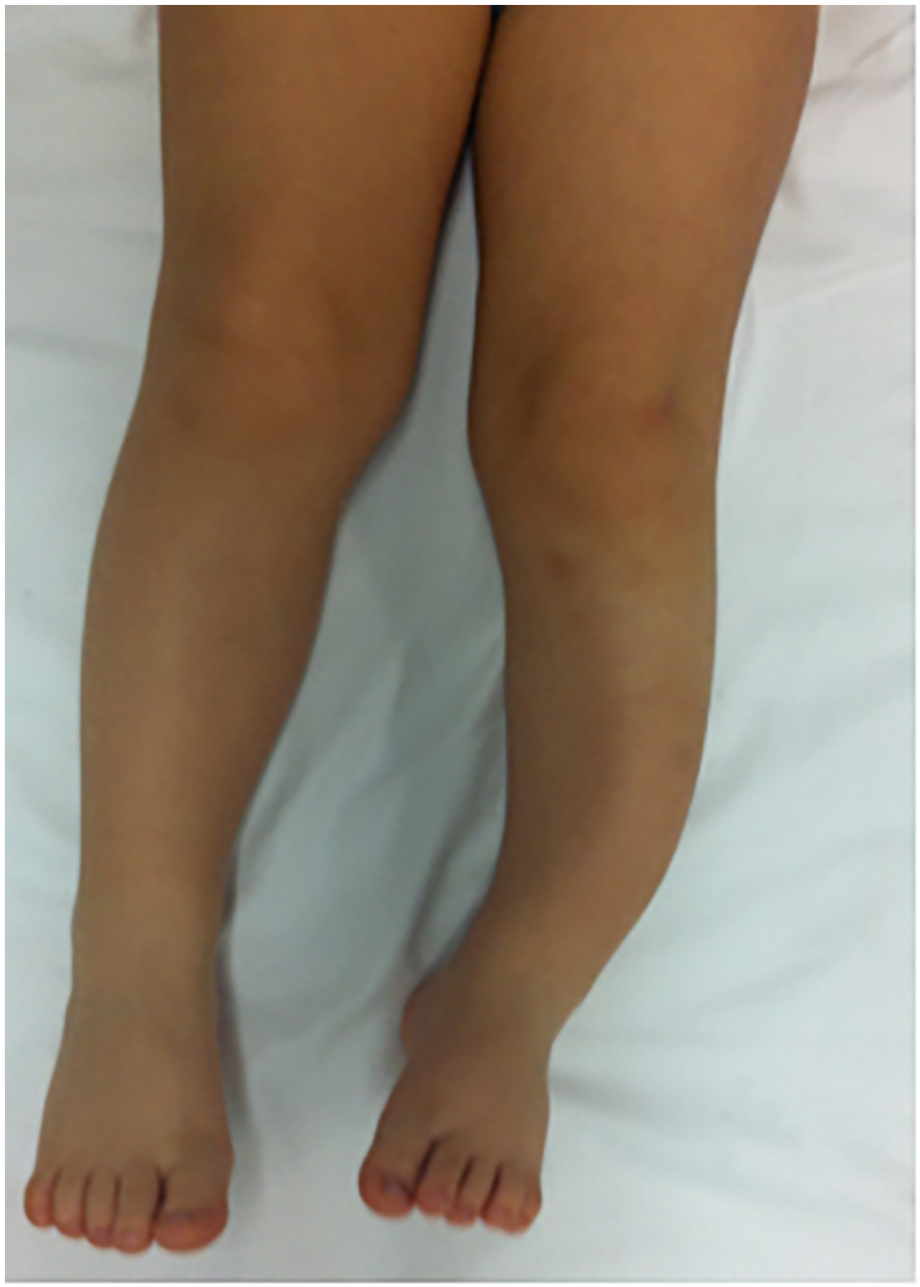
Congenital curvature malformation of the distal third portion of the left leg of a three and a half year old child. Anterolateral type of congenital tibial bowing. Leg-length discrepancy is present.

**Figure 2:**
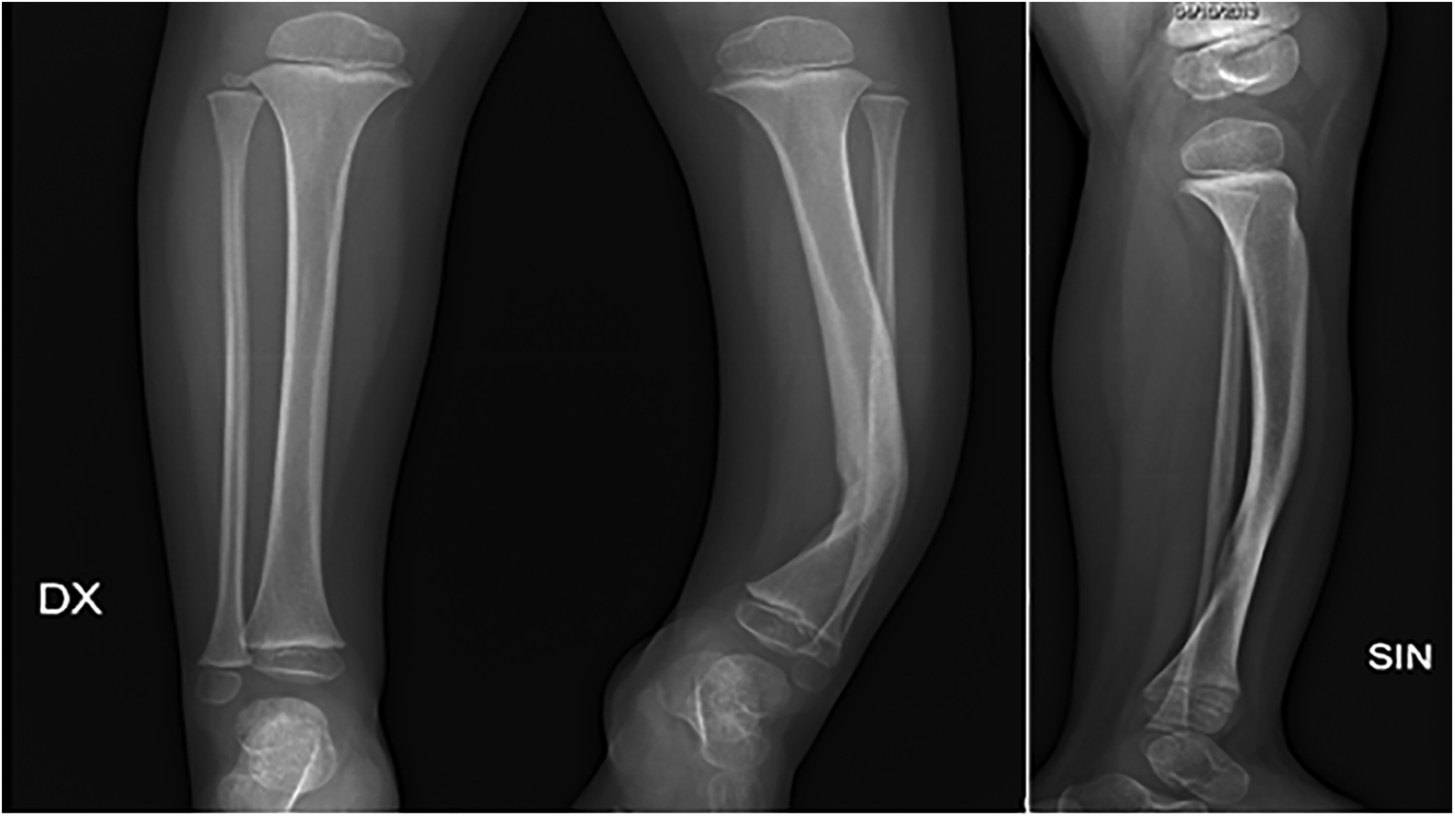
Initial x-rays (AP and LL view) of the anterolateral congenital tibial curvature.

We describe a case of a young child affected by an isolated curvature of his left leg (Figure 1). The congenital malformation was discovered at birth and no other pathological clinical finding was present. The first x-Ray (Figure 2) showed the presence of a congenital curvature of the tibia of the anterolateral type. He was born in another country (Romania) and when he first came to our clinical observation at the Orthopedic and Traumatology Department, Pediatric Hospital “Bambino Gesu” in Rome, the child was 14 months of age and had already started walking. Only a leg-length discrepancy of about 2 cm was present with consequent pelvis obliquity.

**Figure 3:**
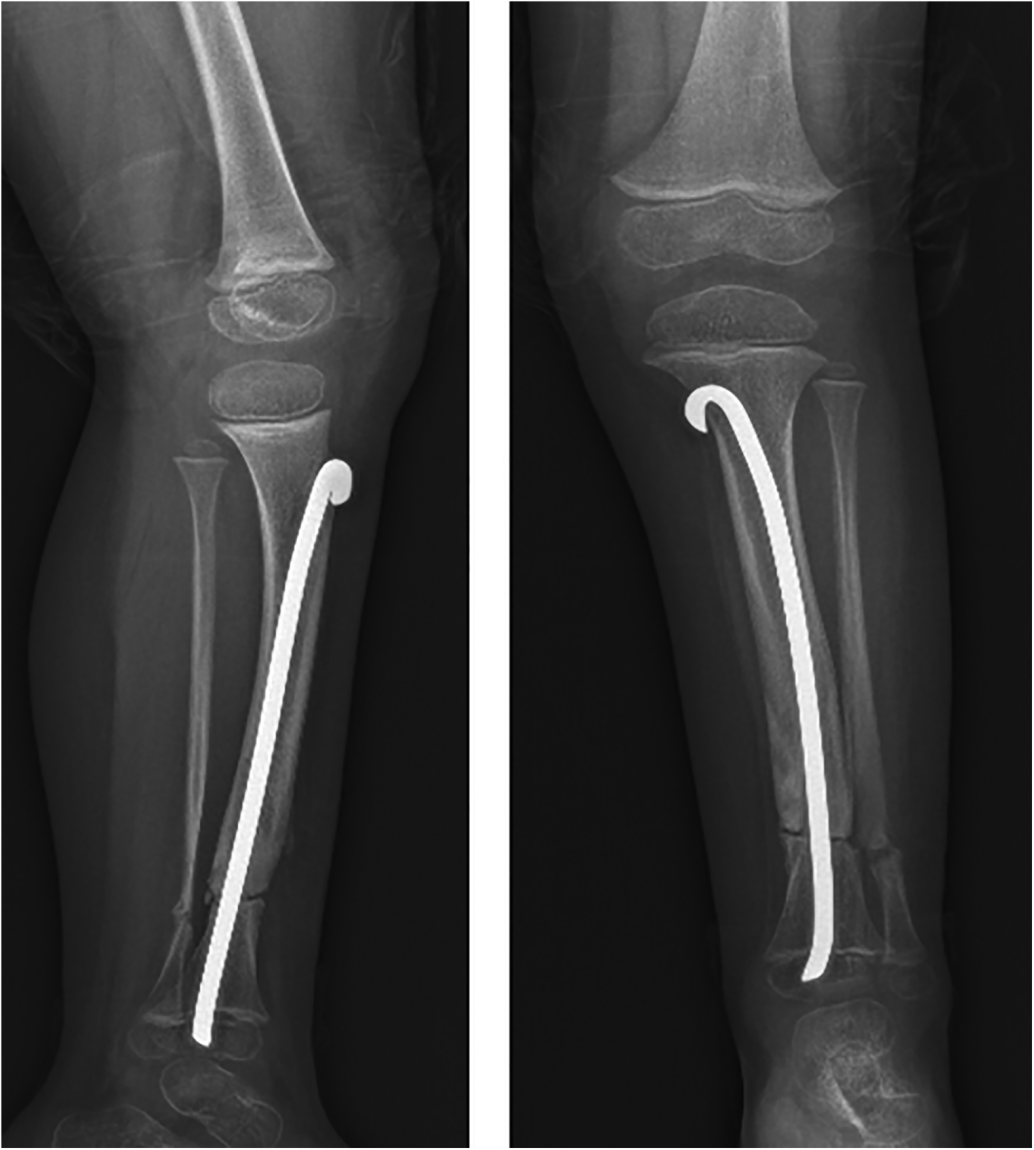
X-ray that shows the rush rod inserted across the distal tibial cartilage growth plate, preserving the ankle joint. Tibial and fibular osteotomy sites are evident.

In the beginning, we prescribed an external clamshell left limb orthoses and a simple shoe lift to prevent a tibial pathological fracture and reduce pelvic obliquity. After several months, despite the external orthoses, at a periodical clinical orthopedic follow-up visit, the child started to show clinical signs and symptoms such as pain and limping on his left leg together with an objective worsening of his congenital tibial curvature. The child's pain was primarily focused on the major convexity of the curvature of his left leg where we observed a little swelling beginning and his joints, left knee, and ankle were starting to suffer, for obvious biomechanical reasons, causing his limping. He did not manifest low-back pain and his leg-length discrepancy appeared to have worsened to about 2.5 cm. At that point, we understood that the child was headed towards the “pre-fracture stage” of his congenital severe tibial curvature and together with his parents we decided to perform surgery to prevent the pathological fracture (Figure 3). At the time of surgery, the child was three and a half years old.

Surgery consisted of a double osteotomy, both of the fibula and the tibia. Subtraction of the distal meta-diaphyseal portion of the fibula and tibial osteotomy in correspondence with the major anterolateral curvature. The tibial osteotomy was then stabilized by an internal Rush rod inserted proximally to the tibia under the cartilage growth plate and made it end inside the distal tibial epiphysis, crossing the distal tibial cartilage growth plate and preserving the ankle joint. The Rush rod was modified with a slight curvature of its distal portion to allow the correct proximal insertion and stabilization of the tibial osteotomy site.

After surgery, the child's left leg was immobilized with a “femur-foot cast” for 50 days and then with a “boot cast” for a further 30 days. After this period, we again prescribed external clamshell orthoses for the beginning of the walking phase. The child maintained the external orthoses for approximately one year from surgery until the osteotomy site appeared stable with an x-Ray showing advanced tibial bone healing process. The child was only allowed to swim without diving and was not allowed to jump or run for the first year after surgery.

## Results

**Figure 4:**
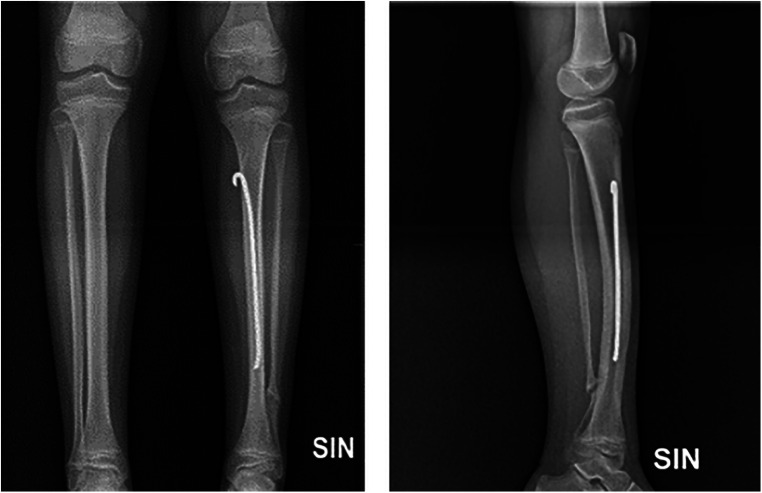
X-ray after eight-year follow-up. The rush rod is far away from the distal tibial cartilage growth plate. The tibial osteotomy site is perfectly healed. No growth plate disturbances are present.

The patient a few months after surgery and from cast removal soon had an excellent clinical outcome. The tibial osteotomy site healed slowly and after six months from surgery was perfectly healed. At periodical orthopedic follow-up visits, it was found always to be better. Any clinical significative evidence of growth disturbances, due to the Rush rod inserted across the distal tibial cartilage growth plate, was noted; rather, x-rays showed that the Rush rod progressively migrated upwards together with tibial growth. Moreover, even the leg-length discrepancy and the pelvic obliquity improved, from the initial 2.5 cm to 0.5 cm. After eight years of follow-up, the patient, now a young boy of 11 and a half years, has an excellent clinical outcome without pain or lower limb dysfunction (Figure 4). We still did not remove the Rush rod because we were always afraid of having to deal with an unpredictable tibial pathological fracture, but we are now seriously considering removing it soon.

## Discussion

Congenital pseudarthrosis of the tibia is a rare and challenging pediatric condition. The “pre-fracture stage”, called congenital tibial dysplasia or anterolateral bowing of the tibia, presents a high fracture risk due to underlying bowing and dysplasia. After a fracture, there is a substantial risk of non-union (8, 9). Any union achieved may be complicated by re-fracture, deformity, leg-length discrepancy, stiffness, pain, and dysfunction (8, 9, 21).

The primary consideration in the treatment of CPT is the expected union rate and re-fracture risk. Combined intramedullary and external or internal fixation, especially with cross-union techniques, show the most promise. Perhaps most exciting is further research on preventing fractures through guided growth, which may reduce the morbidity of multiple surgical procedures which have been the mainstay of treatment for CPT thus far (19–22).

Our case report surely contributes further important information for the treatment and management of these rare congenital disorders. In particular, it prevented tibial fracture or pseudarthrosis, decreased tibial malalignment, improved the radiographic appearance of bone quality, and improved leg-length discrepancy. No growth disturbances were observed for the Rush rod inserted across the distal tibial cartilage growth plate. Instead, the preservation of the ankle joint from the Rush rod insertion determined a normal ankle joint, avoiding dealing with other problems such as stiffness, limping, chronic inflammation, and weight-bearing pain and dysfunction.

We modified the distal portion of a Rush rod, giving it a slight curvature that allowed us to proceed in a correct surgical manner for its proximal tibial insertion under the tibial cartilage growth plate. The Rush rod was then inserted into the endomedullary tibia, with a proximal-distally direction. The rod crossed the tibial osteotomy site and we made it end inside the distal tibial epiphysis, crossing the distal tibial cartilage growth plate, preserving the ankle joint.

## Conclusion

Despite the rarity of this condition, a lot of work has been done in this area.

Initial treatment of tibial bowing deformity includes stretching, serial casting, or splinting. Indication for a tibial osteotomy may be justified only in a significant deformity that interferes with growth, especially in young patients, if no correction is obtained by the age of two years or if symptomatic and severe deformity persists (17).

Several recent studies on the surgical treatment for CPT are now available in the literature that are showing encouraging results, such as: “Congenital pseudarthrosis of the tibia: Rate of and time to bone union following contralateral vascularized periosteal tibial graft transplantation” (15); the “Cross-Union Technique” (8); “Distal Tibial Guided Growth for Anterolateral Bowing of the Tibia: Fracture may be prevented” (21); and “Does An Osteotomy Performed in Congentital Pseudarthrosis of the Tibia Heal?” (19).

In our case report, no metal changes due to the steel Rush rod utilized for the intramedullary tibial nailing were observed and, as mentioned above, we are seriously considering removing it soon. In addition, we did not use other adjunctive treatment options like preoperative bisphosphonates and/or BMP-2, as reported in recent papers (19).

We suggest the method described in this paper only for severe cases of congenital tibial curvatures that clinically appear to undergo a pathological fracture that can lead to CPT.

The surgical treatment method described gave us an excellent outcome at an eight-year follow-up, even if we had to wait, in the beginning, about six months to achieve a good bone union at the osteotomy tibial site, which was perfectly in line with recent papers (19).

All these techniques represent new tools for the surgical treatment of these congenital rare disorders and recent papers are showing excellent outcomes in obtaining bone union and reducing amputation rates for these conditions.

We are all confident and hopeful for the other existing surgical trends that are all showing excellent outcomes for CPT and its complications, achieving bone union, reducing re-fracture and the morbidity of multiple surgical procedures, and not least the decreasing of the percentage of amputation rate (15, 19, 20).

Altogether, this undoubtedly provides much information to all pediatric orthopedic surgeons who deal with such biologically complex situations and who can finally start choosing the best reconstructive strategy in each of these singular congenital rare cases.

Our case report described together with the relative surgical technique performed could be considered as an initial surgical treatment option for these severe anterolateral congenital tibial curvatures during the “pre-fracture stage” and that all the other surgical treatments available can be considered in later phases.

The management of the “pre-fracture stage” in severe congenital tibial curvature disorder together with the surgical technique performed, as described in our case report, to our knowledge, has never been reported in the literature.

It produced a satisfactory long-term functional outcome and adds further important information to keep in mind for the management of such congenital rare disorders, even during the “pre-pathological fracture stage”.

Written informed consent for the publication of this case report, with all data and images, was obtained from the child's parents, and the Scientific Committee of “Bambino Gesu’” Pediatric Hospital, Rome, gave the approval for its publication.

## Data Availability

The original contributions presented in the study are included in the article, further inquiries can be directed to the corresponding author.

## References

[1] Han J Qu L Li Y Luo J Cao J Zhao W. Review: a benign form of congenital anterolateral bowing of the tibia associated with ipsilateral polydactyly of the hallux: case report and literature review. Am J Med Genet A. (2012) 158A(7):1742–9. 10.1002/ajmg.a.35417 22678991

[2] Grill F Bollini G Dungl P Fixsen J Hefti F Ippolito E Treatment approaches for congenital pseudarthrosis of the tibia: results of the EPOS multicenter study. J Pediatric Orthop B. (2000) 9(2):75–89. 10.1097/01202412-200004000-00002 10868356

[3] Heyman CH Herndon CH. Congenital posterior angulation of the tibia. J Bone Joint Surg Am. (1949) 31A(3):571–80. 10.2106/00004623-194931030-00014 18153899

[4] Judet J Judet R Rigault P Roy-Camille R. Treatment of congenital pseudarthrosis of the leg by decortication, external fixator and secondary reinforcement graft. Rev Chir Orthop Reparatrice Appar Mot. (1968) 54(6):503–10. 4236109

[5] Tuncay IC Johnston CE 2nd Birch JG. Spontaneous resolution of congenital anterolateral bowing of the tibia. J Pediatr Orthop. (1994) 14(5):599–602. 10.1097/01241398-199409000-00008 7962500

[6] Herring JA. Tachdjian’s Pediatric Orthopaedics, from the Texas Scottish Rite Hospital for Children E-Book. 3 edn. Vol 2. Texas Scottish Rite Hospital for Children: John Anthony Herring (2002). p. 867–83.

[7] Crawford AH Schorry EK. Neurofibromatosis in children: the role of the orthopaedist. J Am Acad Orthop Surg. (1999) 7(4):217–30. 10.5435/00124635-199907000-00002 10434076

[8] Shannon CE Huser AJ Paley D. Cross-union surgery for congenital pseudarthrosis of the tibia. Children. (2021) 8(7):547. 10.3390/children8070547 34202921 PMC8303361

[9] Paley D. Congenital pseudarthrosis of the tibia: biological and biomechanical considerations to achieve union and prevent refracture. J Child Orthop. (2019) 13(2):120–33. 10.1302/1863-2548.13.180147 30996736 PMC6442511

[10] McClure PK Franzone JM Herzenberg JE. Challenges with fassier-duval rod exchanges in congenital pseudarthrosis of the tibia: explant roadblock and solution. J Pediatr Orthop B. (2022) 31(1):e95–e100. 10.1097/BPB.0000000000000907 34380988

[11] Agrawal U Tiwari V. Congenital tibial pseudarthrosis. In: Statpearls treasure Island. FL: StatPearls (2023). 35015468

[12] Joseph B Somaraju VVJ Shetty SK. Management of congenital pseudarthrosis of the tibia in children under 3 years of age. Effect of early surgery on union of pseudarthrosis and growth of the limb”. J Pediatric Orthop. (2003) 23(6):740–6. 10.1097/01241398-200311000-00011 14581777

[13] Boyd HB. Pathology and natural history of congenital pseudarthrosis of the tibia. Clin Orthop Relat Res. (1982) 166:5–13. 7083685

[14] Codivilla A. On the cure of the congenital pseudoarthrosis of the tibia by means of periosteal transplantation. Archive. Am J Orthop Surg. (1906) s2-4(2):163–9.

[15] Soldado F Barrera-Ochoa S Romero-Larrauri P Nguyen T-Q Diaz-Gallardo P Guerra E Congenital pseudarthrosis of the tibia: rate and time to bone union following contralateral vascularized periosteal tibial graft transplantation. Microsurgery. (2022) 42(4):326–32. 10.1002/micr.30868 35137443

[16] Guidera KJ Raney EM Ganey T Albani W Pugh L Ogden JA. Ilizarov treatment of congenital pseudathroses of the tibia. J Paediatric Ortop. (1997) 17(5):668–74. 10.1097/01241398-199709000-00018 9592009

[17] McCarthy JJ. Chief Editor: DeBerardino TM. Tibial Bowing Treatment and Management. Medscape Reference. Updated may 04, 2022. emedicine – Medscape, article 1251215.

[18] Keriseddy N Kheireldin R Lu A Cooper J Liu J Ebraheim NA. Current treatment of congenital pseudarthrosis of the tibia: a systematic review and meta-analysis. J Paediatric Ortop B. (2018) 27(6):541–50. 10.1097/BPB.0000000000000524 29878977

[19] Nahm NJ Makarevich CA Rosenwasser KA Herzenberg JE McClure PK. Does an osteotomy performed in congentital pseudarthrosis of the tibia heal? J Pediatric Orthop. (2022) 42(6):e630–5. 10.1097/BPO.0000000000002148 35348473

[20] Siebert MJ Makarewich CA. Anterolateral tibial bowing and congenital pseudathrosis of the tibia: current concept review and future directions. Curr Rev Musculoskel Med. (2022) 15(6):438–46. 10.1007/s12178-022-09779-y PMC9789274 35841513

[21] Laine JC Novotny SA Weber EW Georgiadis AG Dahl MT. Distal tibial guided growth for anterolateral bowing of the tibia: fracture may be prevented. J Bone Joint Surg Am. (2020) 102(23):2077–86. 10.2106/JBJS.20.00657 33093298

[22] Patel M. Chief Editor: DeBerardino TM. Tibial Nonunions. Medscape Reference. Updated Sep 12, 2022. emedicine.

